# EFG‐CS: Predicting chemical shifts from amino acid sequences with protein structure prediction using machine learning and deep learning models

**DOI:** 10.1002/pro.5096

**Published:** 2024-07-09

**Authors:** Xiaotong Gu, Yoochan Myung, Carlos H. M. Rodrigues, David B. Ascher

**Affiliations:** ^1^ The Australian Centre for Ecogenomics, School of Chemistry and Molecular Biosciences University of Queensland Brisbane Queensland Australia; ^2^ Computational Biology and Clinical Informatics Baker Heart and Diabetes Institute Melbourne Victoria Australia

**Keywords:** chemical shift, deep learning, machine learning, protein prediction, web server

## Abstract

Nuclear magnetic resonance (NMR) crystallography is one of the main methods in structural biology for analyzing protein stereochemistry and structure. The chemical shift of the resonance frequency reflects the effect of the protons in a molecule producing distinct NMR signals in different chemical environments. Apprehending chemical shifts from NMR signals can be challenging since having an NMR structure does not necessarily provide all the required chemical shift information, making predictive models essential for accurately deducing chemical shifts, either from protein structures or, more ideally, directly from amino acid sequences. Here, we present EFG‐CS, a web server that specializes in chemical shift prediction. EFG‐CS employs a machine learning‐based transfer prediction model for backbone atom chemical shift prediction, using ESMFold‐predicted protein structures. Additionally, ESG‐CS incorporates a graph neural network‐based model to provide comprehensive side‐chain atom chemical shift predictions. Our method demonstrated reliable performance in backbone atom prediction, achieving comparable accuracy levels with root mean square errors (RMSE) of 0.30 ppm for H, 0.22 ppm for Hα, 0.89 ppm for C, 0.89 ppm for Cα, 0.84 ppm for Cβ, and 1.69 ppm for N. Moreover, our approach also showed predictive capabilities in side‐chain atom chemical shift prediction achieving RMSE values of 0.71 ppm for Hβ, 0.74–1.15 ppm for Hδ, and 0.58–0.94 ppm for Hγ, solely utilizing amino acid sequences without homology or feature curation. This work shows for the first time that generative AI protein models can predict NMR shifts nearly comparable to experimental models. This web server is freely available at https://biosig.lab.uq.edu.au/efg_cs, and the chemical shift prediction results can be downloaded in tabular format and visualized in 3D format.

## 1 INTRODUCTION

Nuclear magnetic resonance (NMR) spectroscopy is the combined experimental science of solid‐state NMR spectroscopy and x‐ray diffraction. The advantages that make NMR outstanding compared with other spectroscopic techniques include its nondestructiveness and informativeness in explicating molecular structure. The chemical shift of the resonance frequency, or simply chemical shift, is the effect of the protons in the molecule producing different NMR signals with different chemical environments. It is closely associated with the structural information of compounds, for instance, the conformations of backbone and side‐chain atoms. High‐accuracy chemical shifts in NMR spectra for large molecules like proteins are influenced by factors such as protein secondary structure (Wang and Jardetzky, 2002), solvent exposure of amino acid residues (Vranken and Rieping, 2009), and estimation of backbone torsion angles (Shen et al., 2009). While chemical shift can be utilized to derive 3D protein structures, various factors, for example, the local electric fields and electronic circulation, hydrogen bonding, as well as ring‐current effects, make it challenging to use deterministic evaluation of chemical shift values.

It has been well recognized that chemical shifts exhibit a strong correlation with the structural characteristics of proteins, particularly the molecular framework of their structures. Comparatively accurate modules have been developed to utilize chemical shifts for determining backbone dihedral angle values in the field of NMR (Shen et al., 2009). However, to fully utilize and extend the prediction of complete protein tertiary structure, it is crucial to predict the corresponding chemical shifts for a given protein structure. Chemical shift prediction has been proven to have substantial potential in resolving crystal structures for applications in molecular crystals, biomolecules, and materials (Ashbrook and McKay, 2016). The fundamental challenge in acquiring secondary and tertiary structural information for large molecules involves accurately mapping the complex, nonlinear relationship between chemical shift values and structure. While various empirical optimisation methods show promising results (Hu et al., 2021), this challenge remains a critical aspect of ongoing research focus in multiple domains. Existing chemical shift prediction techniques depend upon large‐scale experimental datasets along with practical heuristics that enable the rapid, albeit less rigorous, simulation of protein chemical shifts.

Chemical shift prediction in NMR requires standardized molecular training and test sets for accurate evaluation and benchmarking. The Biological Magnetic Resonance Bank (BMRB) database (Hoch et al., 2023) is a commonly used resource for this purpose, providing experimentally determined NMR chemical shifts for a diverse range of biomolecules including proteins, nucleic acids, and carbohydrates. Another widely used set is the SHIFTX2 (Neal et al., 2003) database, which contains experimentally determined backbone and side‐chain NMR chemical shifts for proteins. With increasing data sizes, database‐derived methods with the capability of analyzing resulting data are also making substantial progress. This progress is particularly evident in the enhanced ability to extract and utilize intricate protein structural information. Prominent techniques including the prominent techniques of SHIFTX2 (Neal et al., 2003), Camshift (Kohlhoff et al., 2009), SPARTA+ (Shen et al., 2009), CASPER (Lundborg and Widmalm, 2011), and PPM (Li and Brüschweiler, 2012) have benefited from the enrichment of datasets. These techniques now offer more refined predictions of protein structural and chemical properties, providing researchers in different areas with indispensable insights into molecular behavior, interactions, and flexibilities. Also, these datasets enable rigorous evaluation and facilitate the development of more robust approaches for NMR spectroscopy.

The variations of predicted chemical shift results with computational models should be close to the experimental variations between diverse structures so that the models can be useful for structure elucidation. The accuracy rates of most previously designed methods were below that of Density Functional Theory (DFT) calculations. Unzueta et al. (2021) presented a ∆‐machine learning (ML) approach of an ensemble of neural networks that achieved the errors for chemical shielding compared to the anticipated deviations from DFT chemical shifts with respect to experimental values, often achieving just one‐half to one‐third of the expected differences. Kang et al. (2020) proposed a weakly supervised ML model for chemical shift prediction with only molecular level annotation that achieved comparable results to the fully supervised methods of ^1^H and ^13^C spectra prediction. Cordova et al. (2022), the team behind the development of the ShiftML machine learning model for predicting chemical shifts in molecular solids, expanded its capabilities to predict chemical shifts for a broader range of chemically diverse compounds and finite temperature structures. The updated ShiftML2 (Cordova et al., 2022) achieved a comparable accuracy rate with dramatically reduced computational cost, particularly for distorted structures, within a benchmark set of 13 molecular solids.

Like many other challenging problems in computational biology, computational resources required for chemical approaches can be substantial, especially for conformationally flexible large molecules (Wu et al., 2017). Despite advancements providing easier access to high‐performance computational resources and the progress of quantum mechanical methods, the complexities persist. Although being routinely used, empirical approaches that utilize 2D molecular graphs have their limitations, since the molecular conformation is not appropriately captured by common atomic environments description that encodes local connectivity. Guan et al. (2021) stated that models which utilize spatial representations of the atomic environment in 3D molecular graph formation can address this challenge, as bonded and nonbonded interactions of interatomic distances can capture chemical shift variations across diastereoisomeric molecules, spatially distinct conformations of molecules, and diastereotopic groups of a molecule. Utilizing experimentally measured chemical shift datasets, empirical approaches of ML methods for chemical shift prediction calculate electronic structure relying on feature engineering with expert‐crafted rules (Gallegos et al., 2021), while some deep learning approaches, for instance, graph neural networks (GNNs) with embodied feature learning capacity enable “end‐to‐end” learning (Guan et al., 2021) from molecular structures and avoid rule‐based feature encoding. GNNs are a type of neural network that can operate on graph‐structured data, such as protein structures represented as graphs. GNNs have become a common approach for molecule predictions due to their flexibility in molecular graph specification and their intuitive connection to molecular graphs (Cai et al., 2022; Yang et al., 2021). GNNs have also been applied to various protein‐related tasks, such as protein‐ligand binding prediction (Jiang et al., 2022), protein contact prediction (Jha et al., 2022), and mutations on protein stability (Wang et al., 2023). Large complexes' structure determination based on experimental NMR data is challenging because of the complexity of calculations, inspiring Wright et al. (2020) to present a GPU‐accelerated approach to estimate and calculate chemical shift prediction named PPM_One. Han et al. (2022) proposed a scalable GNN sparsifying the graph representation of molecules by only considering heavy atoms as nodes and the relevant chemical bonds as edges. They also improved message passing by adapting the attention mechanism as well as using node‐level and graph‐level embeddings as the readout functions to achieve higher chemical shift prediction accuracy.

Chemical shift prediction from amino acid sequences is a challenging problem in computational biology. Relying on the experimental protein structure for NMR chemical shifts prediction could be a prolonged waiting for the progress of scientific breakthroughs. The Protein Data Bank (PDB) currently archived more than 175,000 experimentally determined 3D protein structures (Velankar et al., 2021), and the number is constantly growing with the contribution of scientist worldwide. However, the experimental results represent a small portion of the billions of currently known protein sequences (Jumper et al., 2021). The total number of proteins in existence on earth is theoretically infinite because of the diverse combination and modification of amino acids and difficult to be precisely determined due to the complexity and vastness of the proteome. Several computational methods have been developed for predicting chemical shifts, including empirical methods based on sequence and structural features, quantum mechanics calculations, and ML approaches. Empirical methods such as SHIFTX2 (Neal et al., 2003) and SPARTA+ (Shen et al., 2009) use sequence‐derived features such as amino acid type, secondary structure, and solvent accessibility to predict chemical shifts. Quantum mechanics calculations can provide accurate chemical shift predictions, but they are computationally expensive and not practical for large‐scale applications.

Accurate amino acid‐based chemical shift prediction can also in return advance the protein structure prediction as chemical shifts are precise probes of secondary structures in protein folding for both ordered and disordered proteins and have main substantially utilized to map protein backbone dihedral angles (Shen and Bax, 2015). Sanz‐Hernández and De Simone (2017) presented a chemical shift calculation approach using exclusively the sequences information of ordered and disordered proteins named sequence‐based approach, protein sequences and chemical shift correlations (PROSECCO). This sequence‐based PROSECCO statistic approach achieved a comparable accuracy rate with advanced structure‐based methods for folded protein chemical shift prediction using Gaussian kernel‐based neighbor correction method. It is important to highlight, as noted by the authors, that the final implementation, PROSECCO_FOLDED_, for successful prediction from amino acid sequences relies on the accuracy of secondary structure prediction, which, in turn, influences the Q3 classification indexing of chemical shifts, which can be enhanced for sequence‐based chemical shift prediction approaches.

Recent advancements in accurate protein structure prediction tools have highlighted the scarcity of protein sequence‐based chemical shift prediction tools. Commencing with the recent revolutionary AlphaFold (Jumper et al., 2021), the first computational model capable of predicting protein structures at atomic precision, significant strides have been made to bridge the gap between approximately 100,000 empirically determined protein structures and the extensive collection of billions of known protein sequences. ESMFold (Lin et al., 2023) is another end‐to‐end Transformer protein language model from the Meta Fundamental AI Research Protein Team (FAIR) that exploits the ESM language model to produce high accuracy predicted protein structure directly from protein amino acid sequences. The performance results of ESMFold match AlphaFold2 when the proteins' perplexity is low, while the prediction speed of ESMFold considerably improved by six times faster on a protein with 384 residues and 60 times faster on shorter sequences than a single AlphaFold2 model (Lin et al., 2023). As most experimentally observed chemical shifts deposited on BMRB do not involve proteins with more than 1000 amino acids, ESMFold is an appropriate choice for protein structure prediction, particularly for its computational efficiency.

While chemical shifts are traditionally obtained via NMR spectroscopy, we present EFG‐CS, a web server poised to revolutionize this norm by computationally predicting chemical shifts from amino acid sequences. Utilizing protein structures predicted by ESMFold, EFG‐CS enables the prediction of both backbone and side‐chain chemical shifts without relying on experimentally determined NMR data. Our platform, EFG‐CS—short for “ESMFold‐Guided Chemical Shifts”—affords users the flexibility to input amino acid sequences or PDB‐format protein structures, thereby accommodating a broad range of research preferences and needs. As there are not many such tools available to the best of our knowledge, it would benefit multiple domain users to provide a platform with the options of utilizing both amino acid sequence and structure for chemical shift prediction. Our platform leverages ESMFold for protein structure prediction, employing a ML‐based transfer model for accurate backbone atom chemical shift prediction and a GNN‐based model for comprehensive side‐chain atom chemical shift prediction. Our platform provides prediction support with outstanding accuracy across a diverse range of domains without the limitations of empirical results or data curation. The web server is freely available at https://biosig.lab.uq.edu.au/efg_cs.

## 2 RESULTS

The performance of our pipeline for chemical shifts in both backbone and side‐chain atoms was independently assessed using two distinct blinded datasets. The GNN model for side‐chain atom prediction was evaluated with the SHIFTX2 test dataset, while the ML‐based model for backbone atom prediction was evaluated across the test dataset of UCBShift (Li et al., 2020) to ensure the test datasets remained uncontaminated by either training datasets.

**FIGURE 1:**
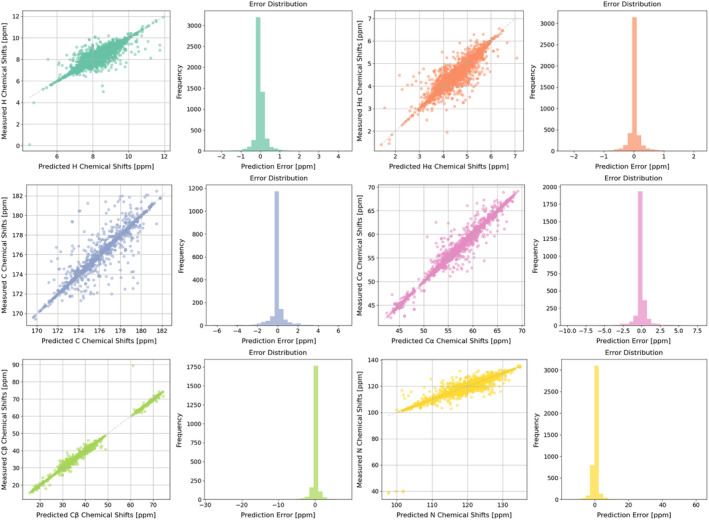
Parity plots showing the correlation between experimentally measured and predicted chemical shifts for backbone atoms (H, Hα, C, Cα, Cβ, N). Each point represents a prediction, with the solid line indicating perfect parity. Deviations from this line illustrate prediction errors, where larger distances indicate greater errors.

The results of backbone atoms evaluation, as shown in Table 1, demonstrate that our pipeline consistently provides accurate predictions of chemical shifts on H, Hα, C, Cα, Cβ, and N atoms across the test dataset. EFG‐CS achieves RMSE values of 0.43 ppm and 0.27 ppm for H and Hα atoms, respectively. Its performance is competitive, closely matching or outperforming SPARTA+ and closely aligning with SHIFTX2 and UCBShift results. EFG‐CS reports higher RMSE values for C (1.52 ppm), Cα (1.13 ppm), and Cβ (1.43 ppm) compared to other tools. These outcomes, while not as low as those based on experimental structures, still reflect a robust capability to predict complex chemical shifts using only predicted structures. N atom prediction is the most challenging, with EFG‐CS registering an RMSE of 2.51 ppm. Though higher than the comparative tools, this value is a significant achievement considering the complexity of accurately modeling nitrogen environments without experimental structure data. The parity plots displayed on the left of Figure 1 demonstrate the correlation between the predicted versus the empirically measured chemical shifts for backbone atoms. This result of data points from the test dataset reveals comparatively strong correlation between the predicted and empirical chemical shifts for these backbone atoms, with relatively minimal deviations. The diagonal alignment, indicating good precision of the pipeline predictions, is consistently achieved for proteins with varying sequences and structures, reinforcing the robustness of our proposed platform. Conversely, the prediction error diagrams positioned on the right of each plot in Figure 1 provide a visual representation of the deviations from the expected values. The prediction error diagrams are important for assessing the spread and distribution of errors across the predictions. For H, Hα, C, Cα, and Cβ, the errors are tightly clustered around zero, indicating minimal deviation from the experimentally measured data. For N atoms, the prediction error diagram reveals a broader spread of error values, mainly due to their complex bonding and insufficient representation in the dataset. These prove that in cases where experimental protein structures are unavailable and data curation is impractical in real‐world scenarios, EFG‐CS can provide accurate backbone atoms chemical shift predictions. This performance is comparable to the tools used for comparison in this study, such as UCBShift, SPARTA+, and SHIFTX2.

**TABLE 1:** Comparison of RMSE values for experimental and predicted backbone atom chemical shifts.

Atom name	SPARTA+	SHIFTX2	UCBShift	EFG‐CS^a^
Hydrogen (H)	0.51	0.44	0.31	0.43
Alpha hydrogen (Hα)	0.27	0.23	0.19	0.27
Carbonyl carbon (C)	1.25	1.16	0.84	1.52
Alpha carbon (Cα)	1.16	1.05	0.81	1.13
Beta carbon (Cβ)	1.35	1.27	1.00	1.43
Nitrogen (N)	2.72	2.40	1.81	2.51

*Note*: The performance is compared among SPARTA+, SHIFTX2, UCBShift, and EFG‐CS across the test dataset of 200 proteins that do not share the same amino acid sequence as the training dataset of backbone atoms prediction. The performance RMSE results are in units of ppm.

Abbreviation: RMSE, root mean square error.

^a^ EFG‐CS utilize ESMFold predicted protein structures, while other tools use experimental protein structures as inputs.

The results of side‐chain atoms assessment shown in Table 2 are the EFG‐CS chemical shift prediction in comparison with NMRGNN results assessed on experimental protein structure reported in their electric supplementary information. This table includes a comprehensive H side‐chain prediction with the atom count over 500 in the SHIFTX2 test dataset. EFG‐CS is evaluated on uncurated dataset without preprocessing of the amino acid sequence input to ensure that the pipeline is evaluated with data that represent the natural usage of our platform. For side‐chain atoms, EFG‐CS shows RMSE values ranging from 0.58 ppm for Hγ 2 up to 0.94 ppm for Hγ 23, indicating relatively consistent performance across different types of hydrogen atoms in side‐chains and aligning with the high‐precision requirements for detailed structural analysis in protein NMR crystallography. The highest RMSE reported by EFG‐CS is 1.26 ppm for He 2, which suggests specific areas where the current model may struggle.

**TABLE 2:** Side‐chain atoms test dataset RMSE between the experimental and the predicted chemical shifts results of NMRGNN and EFG‐CS pipeline.

Atom name	NMRGNN_rmse_	NMRGNN_rmse_ ^a^	EFG‐CS_rsme_
Hβ	0.34	0.68	0.71
Hβ 2	0.32	0.60	0.73
Hβ 3	0.34	0.60	0.79
Hδ 11	0.23	0.57	0.97
Hδ 12	0.23	0.64	0.88
Hδ 13	0.23	0.68	0.94
Hδ 2	0.33	0.85	1.15
Hδ 3	0.30	0.53	0.74
Hε 2	0.25	1.01	1.26
Hγ 2	0.26	0.55	0.58
Hγ 3	0.27	0.53	0.61
Hγ 21	0.26	0.79	0.82
Hγ 22	0.25	0.60	0.94
Hγ 23	0.25	0.65	0.94

*Note*: The performance is compared between NMRGNN and EFG‐CS across the SHIFTX2 test dataset of 61 proteins that do not share the same amino acid sequence as the training dataset of side‐chain atoms prediction. The performance RMSE results are in units of ppm. Beta hydrogen (Hβ), gamma hydrogen (Hγ), delta hydrogen (Hδ), and epsilon (Hε) refer to hydrogen atoms attached to the beta, gamma, delta, and epsilon carbons, respectively. Numbers following Hβ, Hγ, Hδ, and Hε (e.g., Hβ 2, Hγ 21) refer to specific hydrogen atoms on respective carbons when multiple hydrogen atoms are present.

Abbreviation: RMSE, root mean square error.

^a^ Reproduction results of NMRGNN model (Yang et al., 2021) using protein structures determined by x‐ray crystallography, excluding CRYST1 records from the test dataset.

For further insights into the structural accuracy of our models, Table S1 in Data S1 presents the root mean square deviation (RMSD) values calculated between ESMFold‐predicted and experimental protein structures. These RMSD values, computed using PyMOL across all aligned atoms without excluding outliers, provide a quantitative measure of the fidelity of our predicted structures in comparison to their experimentally determined counterparts. While this level of accuracy is generally sufficient for broad applications, we acknowledge that for certain applications requiring finer structural details—like the exact positioning of side‐chains in enzyme active sites or detailed interaction mapping in drug design—lower RMSE values would be more desirable. The side‐chain predictions are inherently more challenging than backbone predictions due to the greater variability in side‐chain conformations, which is reflected in the boarder range of RMSE values observed across different side‐chain atoms.

## 3 DISCUSSION

In this paper, we propose a new platform for predicting chemical shifts from amino acid sequences by harnessing the power of the ESMFold protein folding prediction model and combined with ML and GNN‐based algorithms. ESMFold is a fast and efficient method for generating protein structures from amino acid sequences, which is used to provide input protein structure for the chemical shift prediction models. Our approach is motivated by the recent success of GNNs in predicting protein properties and functions (Yang et al., 2021), as well as the potential of protein folding algorithms to provide structural information for chemical shift prediction.

We evaluated our approach on a dataset of NMR chemical shifts for a diverse set of proteins and showed that it provides accurate chemical shifts prediction. Our results demonstrate the potential of combining GNNs and ESMFold for predicting protein properties and structure from amino acid sequences. This work has important implications for understanding the relationship between protein sequence, structure, and function, and for developing new approaches for predicting protein properties and functions from sequence data. Our work also extends the possibility of using the amino acid sequence with computational models to make accurate chemical shifts prediction that can be utilized in secondary protein structure analysis and to assist NMR experiments to provide the full atlas of protein structures and chemical shifts experimental results.

We believe that our tool contributes to the process of determining protein and protein structures from chemical shifts at a resolution that is comparable to which is provided by standard NMR methods. Align with the current advancement in deep learning‐based protein structure prediction models, we expect our tool can further highlight the improvements in chemical shift‐based structure determination for a wider range of proteins with increased speed and accuracy of chemical shift predictions.

Despite these efforts, accurately predicting chemical shifts for proteins in disordered regions or those without well‐defined secondary structures, such as alpha‐helices or beta‐sheets, remains challenging. Additionally, interactions of proteins with organic substrates can complicate the chemical environment of amino acids. Our model may exhibit variances in these complex scenarios. Features such as post‐translational modifications and areas of high conformational flexibility can influence chemical shifts independently of the backbone conformation and may not be fully addressed in this study. Users should exercise caution when applying our predictions in precise research applications. Moving forward, we plan to enhance our model's robustness and ability to generalize across a broader spectrum of protein features and conditions, focusing particularly on these challenging scenarios.

## 4 MATERIALS AND METHODS

### 4.1 Datasets

The ML‐based transfer model and the graph‐based neural network model were independently trained and tested using distinct datasets to predict chemical shifts for both backbone and side‐chain atoms. Specifically, the ML‐based transfer model was trained using a comprehensive dataset amalgamated from diverse sources. This dataset encompassed the training dataset of SPARTA+ as well as the training and testing datasets of SHIFTX+ (Han et al., 2011). Additionally, the RefDB database (Zhang et al., 2003), containing re‐referenced protein chemical shifts from BMRB, was incorporated to facilitate alignment‐based prediction techniques. The protein structure files sourced from the Structure Bioinformatics Protein Databank (Berman et al., 2000) for training underwent preprocessing, involving the removal of hydrogen atoms and subsequent hydrogen atom addition with Reduce (Word et al., 1999) to ensure alignment with data consistency. Additional preprocessing steps incorporated the exclusion of residues with chemical shift values deviating by more than 5 standard deviations from the mean and those for which DSSP (Joosten et al., 2010; Kabsch and Sander, 1983) failed to generate secondary structure annotations. Residues lacking recorded chemical shifts were systematically removed, along with the exclusion of datapoints exhibiting duplication or involvement of multiple chains.

The GNN model (Yang et al., 2021) undergoes training employing three distinct datasets: the RefDB dataset, an assemblage of cross‐referenced protein structures integrated with their respective chemical shifts; the SHIFTX2 dataset (Han et al., 2011); and the HMDB 4.0 dataset (Wishart et al., 2018) comprising organic molecules. The RefDB dataset encompasses a compendium of 2405 proteins characterized by x‐ray‐resolved crystal structures, encompassing an extensive totality of 131,015,256 atoms and approximately 1.25 million chemical shifts. During preprocessing of the RefDB dataset, each residue within every protein is transformed into a fragment. Fragments presenting with missing residues are excluded, and corrective measures are undertaken for missing atoms. Extraneous components such as solvents and heteroatoms are meticulously eliminated. Additional steps involve ensuring the congruence of NMR chemical shift alignments with the corresponding x‐ray structures and aligning chains appropriately. The SHIFTX2 dataset comprises 197 proteins that were utilized for training, incorporating C, N, and H chemical shifts. Similar preprocessing procedures as implemented for the RefDB dataset are applied to maintain uniformity and accuracy.

The ML‐based transfer model (Li et al., 2020) undergoes rigorous evaluation using the 200 proteins of the UCBShift test dataset, meticulously selected to ensure non‐redundancy by selecting instances that exhibit dissimilar sequences compared to those encompassed within the training dataset. The GNN model is rigorously assessed using the SHIFTX2 test dataset of 61 proteins and incorporating C, N, and H chemical shifts. Notably, these proteins have been meticulously excluded from the RefDB dataset, a constituent in the training phase of the GNN model. Both testing datasets both meticulously constructed by procuring amino acid sequences from the RCSB Protein Data Bank, and particular attention was accorded to excluding sequences involving multiple chains. Subsequently, the 200 protein sequences for backbone atoms testing of the ML‐based model and the 61 protein sequences for side‐chain atoms testing of the GNN model underwent the protein structure prediction procedure via ESMFold, with the integration of hydrogens facilitated by the Reduce tool. The minimum and maximum lengths of amino acid sequences in our test datasets are 21 and 517, respectively. The deliberate strategy was to maintain the test dataset in an uncurated state without preprocessing, striving for maximal authenticity mirroring real‐world deployment scenarios and the application of our proposed computational pipeline.

### 4.2 EFG‐CS pipeline

**FIGURE 2:**
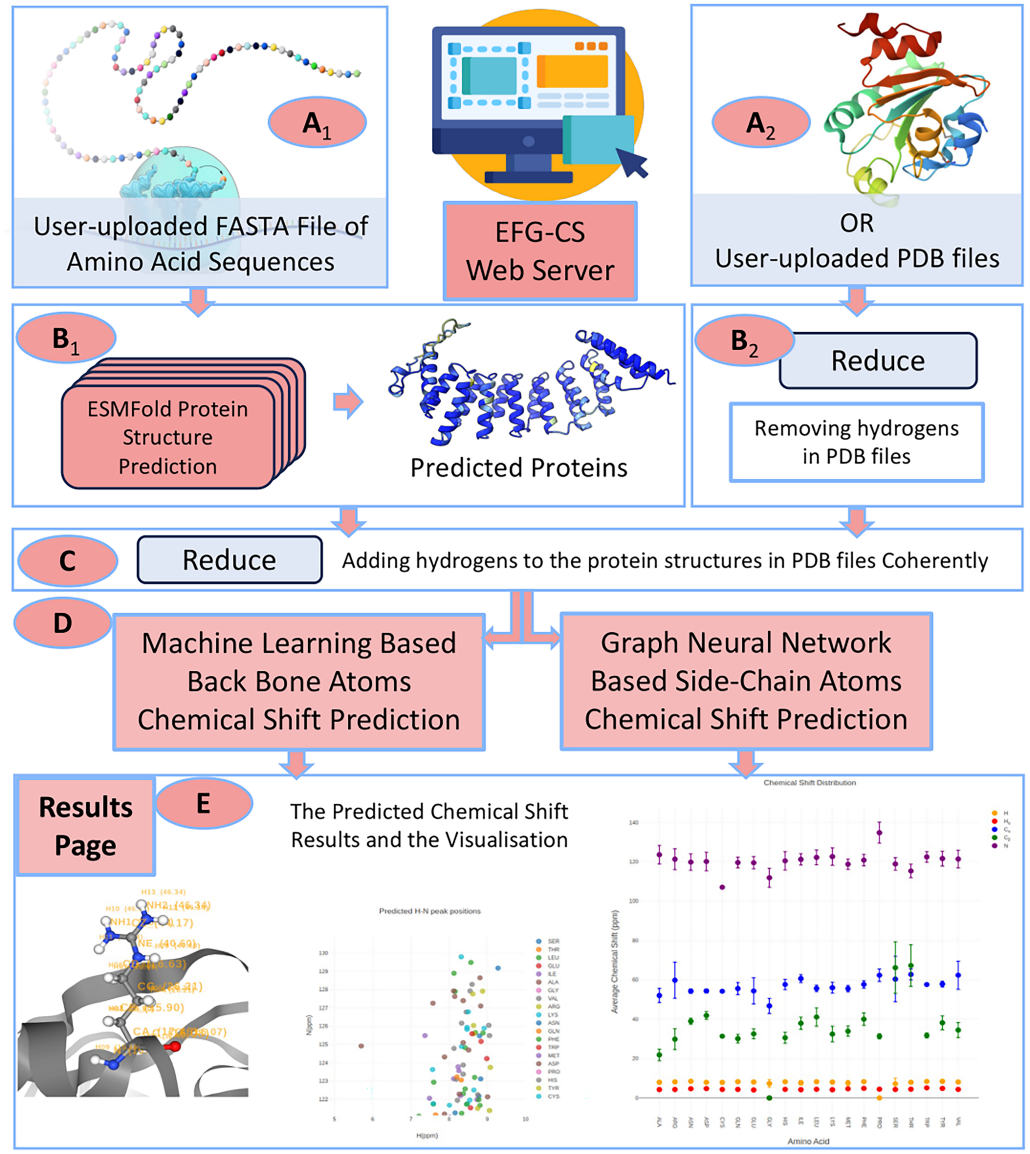
The overall pipeline of EFG‐CS. (a) The presented website for user to provide amino acid sequence(s) (A_1_) or protein structure(s) (A_2_) for chemical shift prediction. (b) The ESMFold model to predict protein structure(s) with confidence from the amino acid sequence(s) (B_1_). Meanwhile, any user‐uploaded protein structures will be processed with the Reduce tool for hydrogen removal (B_2_). (c) Hydrogens are added consistently with Reduce to the protein structure(s). (d) The machine learning‐based model for backbone atom chemical shift prediction and the graph neural network model for side‐chain atom chemical shift prediction from the ESMFold predicted or user‐uploaded protein structure(s). (e) The presented web server on which the user can download the predicted chemical shifts in tabular format and visualize the output results.

The overall architecture of our chemical shift prediction model, termed EFG‐CS and illustrated in Figure 2, combines ESMFold with a ML approach and a GNN. This model consists of five main procedures. First, we utilize ESMFold to produce protein structure prediction for any requested amino acid sequences. The ESMFold predicted protein structure will then be processed by the Reduce tool to add hydrogens before passing to the chemical shift prediction procedure.

For predicting chemical shifts of backbone atoms, we utilize the UCBShift algorithm (Li et al., 2020), an advanced ML‐based approach that leverages both sequence as well as protein structural alignments to transfer empirical chemical shifts from a comprehensive database to target proteins. The UCBShift model is structured with two main components: the transfer prediction module (designated as Y) and the ML‐based module (designated as X). The UCBShift model is then established by combining Y and X along with an additional random forest regressor. The Y module transfers experimental chemical shifts to the query protein when a prominent level of alignment is observed in both the sequence and structure. This is achieved by aligning the amino acid sequences with the RefDB dataset sequences using BLAST (Altschul et al., 1990). Subsequently, the predicted protein structure is aligned using mTM‐align (Dong et al., 2018). Alignments with TM score smaller than 0.8 and RMSD greater than 1.75 Å are appropriately filtered out. The sequence and structure alignment process encompasses three conditions: in cases of identical protein residues, the chemical shifts from RefDB are directly applied to the predicted item; when residues vary, secondary chemical shifts from RefDB are used to consider different shift reference states specific to various amino acids; and if multiple notable structural alignments are present for a residue, an exponential weighting technique is employed to average the secondary chemical shifts from references.

The X model employs features specific to each residue obtained from the protein structure, and these features undergo polynomial transformations. The resulting feature vector from X model's feature extraction is then fed into an extra tree regressor (Geurts et al., 2006) and a subsequent random forest regressor (Breiman, 2001) for generating intermediate predictions. Both regressors partition the data using a subset of features and employ ensemble‐based predictions through a majority vote. In addition to the Y and X models, a second random forest regressor is trained to integrate the secondary shift outputs from the Y model, considering supplementary scores and alignment quality coverage. The final predicting results including independent prediction from both the X and Y model, further enrich the predictive capacity of the overall framework.

For side‐chain atoms chemical shift predictions, we applied a GNN algorithm NMRGNN (Yang et al., 2021), a model that does not require highly curated features while only utilizing the elements of the atoms and distances of the proteins and the residues as input. The model consists of three parts in the architecture: (1) a message passing deep neural network that takes a rank 2 tensor of atom times node feature dimension as the input and produce a same dimension rank 2 tensor as the output; (2) a dense network that takes a rank 3 edge tensor as the input and produce same dimension edge tensor as the output with edge feature dimension; (3) a dense network that produce the chemical shift predictions as the output. The output neighbor features tensor utilize ReLU activation (Glorot et al., 2011) in all but the last layer, in which tanh is used. The predicted chemical shifts *d* of NMRGNN are computed as

$$\vec{\delta }=H\left(V^{k}\right)1_{z}\left(V^{0}\right)\vec{s}+\vec{\mu },$$

where $H\left(V^{k}\right)$ is the dense network in (3) that uses a tanh activation (Karlik and Olgac, 2011) in the penultimate layer, $V^{k}$ is the rank 2 tensor in (1), with the same shape of this neural network's input of atom number multiplied by the node feature dimension, and $\vec{s},\vec{\mu }$ correspond to the predetermined standard deviation and mean, respectively, of the chemical shifts for individual chemical elements extracted from the RefDB dataset. As stated in the original paper (Yang et al., 2021), H exhibits a bias, and with this method, it can produce a chemical shift of 0 for an element that has not been specifically trained.

### 4.3 Web server

We have implemented EFG‐CS as a web server. The front end of web server was developed using Flask 1.0.2 and Materialize 1.0.0, and the back end was built using Python‐3.8. The web server is hosted on an Apache2 Linux server and freely available at https://biosig.lab.uq.edu.au/efg_cs.

#### 4.3.1 Input

EFG‐CS can predict protein chemical shifts from both protein sequence and protein structures. Users with only a protein sequence can submit it through the input tab or by uploading a FASTA file. Alternatively, when utilizing a protein structure, users can upload a PDB file or search and use a structure from the AlphaFold2 database.

#### 4.3.2 Output

EFG‐CS provides the chemical shifts in multiple formats, including a table, 3D viewer, and plot. The predicted backbone chemical shift values are presented in a tabular format, and these values are mapped onto the 3D structure viewer using NGL viewer (Nguyen et al., 2018). Additionally, we rendered a scatter plot illustrating relationships between different atoms and a distribution plot of average chemical shifts for amino acids. Users can interact with the protein visualization to inspect the predicted chemical shift values regarding the atoms of their interests. Users can download the chemical shift results table format.

## AUTHOR CONTRIBUTIONS

**Xiaotong Gu:** Writing – original draft; software; investigation; methodology; formal analysis; validation. **Yoochan Myung:** Writing – review and editing; methodology; software; formal analysis; supervision. **Carlos Henrique Miranda Rodrigues:** Writing – review and editing; software. **David B. Ascher:** Conceptualization; supervision; writing – review and editing; methodology.

## CONFLICT OF INTEREST STATEMENT

The authors declare no conflicts of interest.

## Supporting information


**Data S1.** Supporting information.

## References

[pro5096-bib-0001] Altschul SF , Gish W , Miller W , Myers EW , Lipman DJ . Basic local alignment search tool. J Mol Biol. 1990;215:403–410. 2231712 10.1016/S0022-2836(05)80360-2

[pro5096-bib-0002] Ashbrook SE , McKay D . Combining solid‐state NMR spectroscopy with first‐principles calculations—a guide to NMR crystallography. Chem Commun. 2016;52:7186–7204. 10.1039/c6cc02542k 27117884

[pro5096-bib-0003] Berman HM , Westbrook J , Feng Z , Gilliland G , Bhat TN , Weissig H , et al. The protein data bank. Nucleic Acids Res. 2000;28:235–242. 10592235 10.1093/nar/28.1.235 PMC102472

[pro5096-bib-0004] Breiman L . Random forests. Mach Learn. 2001;45:5–32.

[pro5096-bib-0005] Cai H , Zhang H , Zhao D , Wu J , Wang L . FP‐GNN: a versatile deep learning architecture for enhanced molecular property prediction. Brief Bioinform. 2022;23:bbac408. 36124766 10.1093/bib/bbac408

[pro5096-bib-0006] Cordova M , Engel EA , Stefaniuk A , Paruzzo F , Hofstetter A , Ceriotti M , et al. A machine learning model of chemical shifts for chemically and structurally diverse molecular solids. J Phys Chem C. 2022;126:16710–16720. 10.1021/acs.jpcc.2c03854 PMC9549463 36237276

[pro5096-bib-0007] Dong R , Peng Z , Zhang Y , Yang J . mTM‐align: an algorithm for fast and accurate multiple protein structure alignment. Bioinformatics. 2018;34:1719–1725. 29281009 10.1093/bioinformatics/btx828 PMC5946935

[pro5096-bib-0008] Gallegos LC , Luchini G , St John PC , Kim S , Paton RS . Importance of engineered and learned molecular representations in predicting organic reactivity, selectivity, and chemical properties. Acc Chem Res. 2021;54:827–836. 33534534 10.1021/acs.accounts.0c00745

[pro5096-bib-0009] Geurts P , Ernst D , Wehenkel L . Extremely randomized trees. Mach Learn. 2006;63:3–42.

[pro5096-bib-0010] Glorot X , Bordes A , Bengio Y . Deep sparse rectifier neural networks. Proceedings of the fourteenth international conference on artificial intelligence and statistics. JMLR workshop and conference proceedings. pp. 315–23. 2011.

[pro5096-bib-0011] Guan Y , Sowndarya SS , Gallegos LC , John PCS , Paton RS . Real‐time prediction of ^1^H and ^13^C chemical shifts with DFT accuracy using a 3D graph neural network. Chem Sci. 2021;12:12012–12026. 34667567 10.1039/d1sc03343c PMC8457395

[pro5096-bib-0012] Han B , Liu Y , Ginzinger SW , Wishart DS . SHIFTX2: significantly improved protein chemical shift prediction. J Biomol NMR. 2011;50:43–57. 21448735 10.1007/s10858-011-9478-4 PMC3085061

[pro5096-bib-0013] Han J , Kang H , Kang S , Kwon Y , Lee D , Choi Y‐S . Scalable graph neural network for NMR chemical shift prediction. Phys Chem Chem Phys. 2022;24:26870–26878. 36317530 10.1039/d2cp04542g

[pro5096-bib-0014] Hoch JC , Baskaran K , Burr H , Chin J , Eghbalnia HR , Fujiwara T , et al. Biological magnetic resonance data bank. Nucleic Acids Res. 2023;51:D368–D376. 36478084 10.1093/nar/gkac1050 PMC9825541

[pro5096-bib-0015] Hu YF , Cheng K , He LC , Zhang X , Jiang B , Jiang L , et al. NMR‐based methods for protein analysis. Anal Chem. 2021;93:1866–1879. 33439619 10.1021/acs.analchem.0c03830

[pro5096-bib-0016] Jha K , Saha S , Singh H . Prediction of protein‐protein interaction using graph neural networks. Sci Rep. 2022;12:8360. 35589837 10.1038/s41598-022-12201-9 PMC9120162

[pro5096-bib-0017] Jiang HP , Wang J , Cong WL , Huang YH , Ramezani M , Sarma A , et al. Predicting protein‐ligand docking structure with graph neural network. J Chem Inf Model. 2022;62:2923–2932. 35699430 10.1021/acs.jcim.2c00127 PMC10279412

[pro5096-bib-0018] Joosten RP , Te Beek TA , Krieger E , Hekkelman ML , Hooft RW , Schneider R , et al. A series of PDB related databases for everyday needs. Nucleic Acids Res. 2010;39:D411–D419. 21071423 10.1093/nar/gkq1105 PMC3013697

[pro5096-bib-0019] Jumper J , Evans R , Pritzel A , Green T , Figurnov M , Ronneberger O , et al. Highly accurate protein structure prediction with AlphaFold. Nature. 2021;596:583–589. 34265844 10.1038/s41586-021-03819-2 PMC8371605

[pro5096-bib-0020] Kabsch W , Sander C . Dictionary of protein secondary structure: pattern recognition of hydrogen‐bonded and geometrical features. Biopolymers. 1983;22:2577–2637. 6667333 10.1002/bip.360221211

[pro5096-bib-0021] Kang S , Kwon Y , Lee D , Choi Y‐S . Predictive modeling of NMR chemical shifts without using atomic‐level annotations. J Chem Inf Model. 2020;60:3765–3769. 32692561 10.1021/acs.jcim.0c00494

[pro5096-bib-0022] Karlik B , Olgac AV . Performance analysis of various activation functions in generalized MLP architectures of neural networks. Int J Artif Intell Expert Syst. 2011;1(4):111–122.

[pro5096-bib-0023] Kohlhoff KJ , Robustelli P , Cavalli A , Salvatella X , Vendruscolo M . Fast and accurate predictions of protein NMR chemical shifts from interatomic distances. J Am Chem Soc. 2009;131:13894–13895. 19739624 10.1021/ja903772t

[pro5096-bib-0024] Li D‐W , Brüschweiler R . PPM: a side‐chain and backbone chemical shift predictor for the assessment of protein conformational ensembles. J Biomol NMR. 2012;54:257–265. 22972619 10.1007/s10858-012-9668-8

[pro5096-bib-0025] Li J , Bennett KC , Liu YC , Martin MV , Head‐Gordon T . Accurate prediction of chemical shifts for aqueous protein structure on “real world” data. Chem Sci. 2020;11:3180–3191. 34122823 10.1039/c9sc06561j PMC8152569

[pro5096-bib-0026] Lin Z , Akin H , Rao R , Hie B , Zhu Z , Lu W , et al. Evolutionary‐scale prediction of atomic‐level protein structure with a language model. Science. 2023;379:1123–1130. 36927031 10.1126/science.ade2574

[pro5096-bib-0027] Lundborg M , Widmalm G . Structural analysis of glycans by NMR chemical shift prediction. Anal Chem. 2011;83:1514–1517. 21280662 10.1021/ac1032534

[pro5096-bib-0028] Neal S , Nip AM , Zhang H , Wishart DS . Rapid and accurate calculation of protein ^1^H, ^13^C and ^15^N chemical shifts. J Biomol NMR. 2003;26:215–240. 12766419 10.1023/a:1023812930288

[pro5096-bib-0029] Nguyen H , Case DA , Rose AS . NGLview‐interactive molecular graphics for Jupyter notebooks. Bioinformatics. 2018;34:1241–1242. 29236954 10.1093/bioinformatics/btx789 PMC6031024

[pro5096-bib-0030] Sanz‐Hernández M , De Simone A . The PROSECCO server for chemical shift predictions in ordered and disordered proteins. J Biomol NMR. 2017;69:147–156. 29119515 10.1007/s10858-017-0145-2 PMC5711976

[pro5096-bib-0031] Shen Y , Bax A . Protein structural information derived from NMR chemical shift with the neural network program TALOS‐N. Methods Mol Biol. 2015;1260:17–32. 25502373 10.1007/978-1-4939-2239-0_2 PMC4319698

[pro5096-bib-0032] Shen Y , Delaglio F , Cornilescu G , Bax A . TALOS+: a hybrid method for predicting protein backbone torsion angles from NMR chemical shifts. J Biomol NMR. 2009;44:213–223. 19548092 10.1007/s10858-009-9333-z PMC2726990

[pro5096-bib-0033] Unzueta PA , Greenwell CS , Beran GJ . Predicting density functional theory‐quality nuclear magnetic resonance chemical shifts via δ‐machine learning. J Chem Theory Comput. 2021;17:826–840. 33428408 10.1021/acs.jctc.0c00979

[pro5096-bib-0034] Velankar S , Burley SK , Kurisu G , Hoch JC , Markley JL . The Protein Data Bank archive. Structural proteomics: high‐throughput methods. New York, NY: Humana; 2021. p. 3–21. 10.1007/978-1-0716-1406-8_1 33950382

[pro5096-bib-0035] Vranken WF , Rieping W . Relationship between chemical shift value and accessible surface area for all amino acid atoms. BMC Struct Biol. 2009;9:1–10. 19341463 10.1186/1472-6807-9-20 PMC2678133

[pro5096-bib-0036] Wang SY , Tang HZ , Shan P , Wu ZX , Zuo L . ProS‐GNN: predicting effects of mutations on protein stability using graph neural networks. Comput Biol Chem. 2023;107:107952. 37643501 10.1016/j.compbiolchem.2023.107952

[pro5096-bib-0037] Wang Y , Jardetzky O . Probability‐based protein secondary structure identification using combined NMR chemical‐shift data. Protein Sci. 2002;11:852–861. 11910028 10.1110/ps.3180102 PMC2373532

[pro5096-bib-0038] Wishart DS , Feunang YD , Marcu A , Guo AC , Liang K , Vázquez‐Fresno R , et al. HMDB 4.0: the human metabolome database for 2018. Nucleic Acids Res. 2018;46:D608–D617. 29140435 10.1093/nar/gkx1089 PMC5753273

[pro5096-bib-0039] Word JM , Lovell SC , Richardson JS , Richardson DC . Asparagine and glutamine: using hydrogen atom contacts in the choice of side‐chain amide orientation. J Mol Biol. 1999;285:1735–1747. 9917408 10.1006/jmbi.1998.2401

[pro5096-bib-0040] Wright E , Ferrato MH , Bryer AJ , Searles R , Perilla JR , Chandrasekaran S . Accelerating prediction of chemical shift of protein structures on GPUs: using OpenACC. PLoS Comput Biol. 2020;16:e1007877. 32401799 10.1371/journal.pcbi.1007877 PMC7250467

[pro5096-bib-0041] Wu J , Lorenzo P , Zhong S , Ali M , Butts CP , Myers EL , et al. Synergy of synthesis, computation and NMR reveals correct baulamycin structures. Nature. 2017;547:436–440. 28748934 10.1038/nature23265

[pro5096-bib-0042] Yang Z , Chakraborty M , White AD . Predicting chemical shifts with graph neural networks. Chem Sci. 2021;12:10802–10809. 34476061 10.1039/d1sc01895g PMC8372537

[pro5096-bib-0043] Zhang H , Neal S , Wishart DS . RefDB: a database of uniformly referenced protein chemical shifts. J Biomol NMR. 2003;25:173–195. 12652131 10.1023/a:1022836027055

