# Supplementary material for: EFG‐CS: Predicting chemical shifts from amino acid sequences with protein structure prediction using machine learning and deep learning models
Source: Protein Sci. 2024 Jul 9;33(8):e5096. doi: 10.1002/pro.5096 (PMC11232051; doi:10.1002/pro.5096)
Supplement: Supplementary file 1 — Data S1. Supporting information. [file PRO-33-e5096-s001.docx]

Table S1. Root mean square deviation (RMSD) was calculated between ESMFold-predicted protein structures and experimental protein structures from the SHIFTX2 test dataset, which was used for side-chain chemical shift prediction model evaluation. RMSD was calculated with PyMOL* on all aligned atoms without excluding outliers or superposition.

| **PDB ID** | **RMSD in Angstrom (Å)** |
| --- | --- |
| 1B1H | 1.298 |
| 1BT5 | 0.922 |
| 1CWC | 1.126 |
| 1DQE | 1.701 |
| 1DYT | 1.392 |
| 1H4A | 1.168 |
| 1HQ2 | 1.098 |
| 1HUU | 1.014 |
| 1JIW | 1.634 |
| 1JR2 | 2.757 |
| 1JTG | 1.084 |
| 1KDB | 1.418 |
| 1KF3 | 1.088 |
| 1LM4 | 1.075 |
| 1O4D | 1.201 |
| 1O13 | 1.692 |
| 1ODV | 1.264 |
| 1OQR | 1.219 |
| 1RRO | 2.596 |
| 1RUW | 3.024 |
| 1RWY | 1.191 |
| 1SGZ | 3.344 |
| 1SNM | 1.375 |
| 1SYD | 1.267 |
| 1T2W | 2.307 |
| 1T3Y | 1.636 |
| 1T8L | 1.602 |
| 1T15 | 1.273 |
| 1TP5 | 6.423 |
| 1TP9 | 0.980 |
| 1TVG | 1.367 |
| 1U7B | 1.479 |
| 1UDR | 1.322 |
| 1V9T | 2.036 |
| 1VP6 | 1.294 |
| 1W80 | 1.349 |
| 1XMT | 1.075 |
| 1Y93 | 1.103 |
| 1YJ7 | 9.692 |
| 1YKY | 1.030 |
| 1YSB | 3.058 |
| 1YZ1 | 1.591 |
| 1ZJL | 3.613 |
| 1ZX8 | 1.100 |
| 2A0N | 3.743 |
| 2A38 | 19.719 |
| 2AOJ | 1.192 |
| 2B02 | 2.299 |
| 2BF5 | 5.280 |
| 2C5L | 1.185 |
| 2D3G | 0.872 |
| 2DYI | 3.424 |
| 2ES9 | 2.412 |
| 2ESP | 1.209 |
| 2H30 | 1.138 |
| 2HZE | 1.892 |
| 2IN0 | 1.147 |
| 2O0P | 1.328 |
| 2OYN | 2.375 |
| 2Z2I | 1.541 |
| 256B | 1.208 |

* PyMOL is an open-source molecular visualisation system created by Warren L. DeLano available at <https://pymol.org/.>
